# A Rare Case of Levofloxacin-Induced Acute Pancreatitis: Case Report and Literature Review

**DOI:** 10.7759/cureus.42743

**Published:** 2023-07-31

**Authors:** Osahon Omoregie, Uyonne T Ugwuoke, Osamede Agho, Uwemedimoh C Okonna, Winifred U Iklaki, Grace C Okoro

**Affiliations:** 1 Family and Community Medicine, National Health Service, Glasgow, GBR; 2 Public Health, East Tennessee State University, Johnson City, USA; 3 Internal Medicine, Internal Medicine Solutions, New York, USA; 4 Internal Medicine, Igbinedion University Medical School, Benin, NGA; 5 Obstetrics and Gynecology, American University of Barbados, Chicago, USA; 6 Internal Medicine, All Saints University, School of Medicine, Roseau, DMA; 7 Family Medicine, Brightstar Care, Sugarland, USA

**Keywords:** acute pancreatitis, drug-induced acute pancreatitis, fluoroquinolones, levofloxacin toxicity, severe acute pancreatitis

## Abstract

Diagnosing acute pancreatitis induced by any drug is often overlooked and warrants careful evaluation. Drug-induced acute pancreatitis is relatively rare, and diagnosis of exclusion after ruling out alcohol, gallstones, hypertriglyceridemia, and intervention. Levofloxacin, a class of fluoroquinolones, is generally recommended against various bacterial infections. While levofloxacin is mainly known for its potential side effects, such as photosensitivity and liver toxicity, it can also rarely induce acute pancreatitis. We report a case of acute pancreatitis in a female patient precipitated by levofloxacin. The patient exhibited typical manifestations of acute pancreatitis and had been taking levofloxacin for a urinary tract infection over the past three days. After ruling out other possible causes, her clinical presentation, laboratory results, and imaging findings confirmed levofloxacin-induced acute pancreatitis.

## Introduction

Acute pancreatitis is a severe inflammatory condition characterized by the rapid onset of pancreatic inflammation leading to various complications and potentially life-threatening consequences if not managed promptly [1]. Several factors can trigger acute pancreatitis; gallstone is the most common cause worldwide. These stones can obstruct the pancreatic duct, leading to inflammation. Other causes include alcohol consumption, trauma, medications, viral infections, elevated triglyceride levels, and genetic factors [2]. Drug-induced acute pancreatitis is uncommon but recognized complication of certain medications. Although it is relatively uncommon, it is essential to identify drugs causing acute pancreatitis. Some common causes of drug-induced acute pancreatitis include valproic acid, azathioprine, mercaptopurine, immunomodulatory drugs, thiazide diuretics, macrolides, tetracycline, and sulfonamides [3]. Although rare, levofloxacin has also been reported as an etiology of acute pancreatitis. Only a few cases have been published on levofloxacin as an etiology of acute pancreatitis [4,5]. We underline a case of levofloxacin-induced acute pancreatitis in a female patient.

## Case presentation

A 49-year-old woman with a medical background of celiac disease and type I diabetes mellitus presented with abdominal pain and vomiting over the past 48 hours. The pain was described as aching and crampy, spreading throughout the upper abdomen and extending to the back between the shoulders. It was not alleviated by antacids or food. Additionally, she experienced nausea and had multiple instances of watery, non-bilious projectile vomiting for the past three days. She had recently started taking levofloxacin for the last three days to treat a urinary tract infection. The patient had been compliant with her insulin and amlodipine. There was no history of alcohol consumption, drug abuse, or other medications besides those for diabetes and hypertension. She had not experienced any trauma and had no previous episodes of pancreatitis or similar symptoms.

On examination, she appeared uncomfortable and was clutching her abdomen. She had a blood pressure of 110/70 mmHg, respiratory rate of 20/minute, and heart rate of 95/minute. Her abdomen was tender to palpate in the epigastric region with no rigidity or guarding. Bowel sounds were present, and no visceromegaly was appreciated. The rest of the systemic examination was unremarkable. Her initial laboratory evaluations revealed elevated serum lipase and amylase levels suggestive of acute pancreatitis (Table 1).

**Table 1:** Results of initial laboratory investigations.

Parameter	Lab value	Reference range
White cell count	11000 /mm^3^	(4000-11000)
Hemoglobin	11 g/dl	(13-15)
Bilirubin	0.5 mg/dl	(0.1-1.2)
Amylase	965 IU/L	(30-110)
Lipase	1401 IU/L	(0-155)
Serum creatinine	1.1 mg/dl	(0.7-1.3)
Serum calcium	9.9 mg/dl	(9.0-10)
Blood urea nitrogen	19 mg/dl	(07-26)
Alanine aminotransferase	49 IU/L	(8-57)
Alkaline phosphatase	77 mg/dl	(36-95)
C-reactive protein	3 mg/dl	(0.4-1.1)
Erythrocyte sedimentation rate	31/hour	(<21)

**Figure 1:**
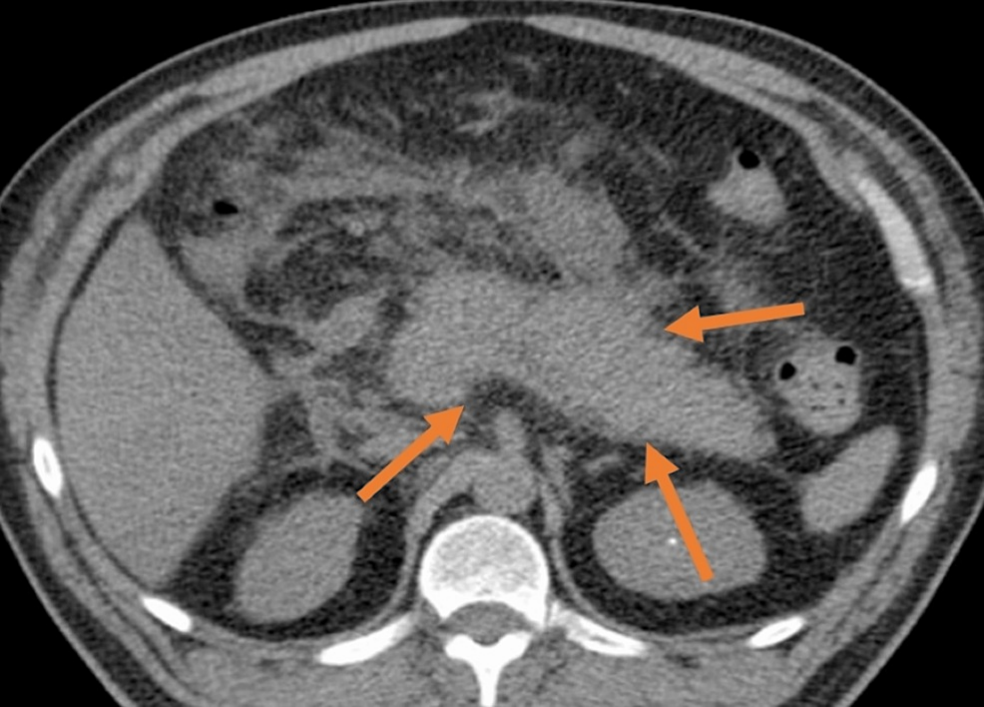
Abdominal computed tomography revealing diffuse pancreatic inflammation with ill-defined borders.

Abdominal ultrasound demonstrated a non-distended gallbladder without wall edema and thickening; the pancreas was not visualized. Abdomen CT was performed, which showed diffuse and generalized enhancement of the pancreas with ill-defined borders and fat stranding, and localized edema. No gallstone, biliary sludge, pseudocyst, or biliary tract abnormality was appreciated (Figure 1). Other lab evaluations were unremarkable, including autoimmune screening, fasting lipid profile, and calcium levels. Viral serology tests involving hepatitis B, C, and human immunodeficiency virus, were negative to rule out an infectious cause of acute pancreatitis. Based on clinical, serological, and imaging modalities, a provisional diagnosis of acute pancreatitis induced by levofloxacin was made after ruling out all other possible causes.

**Figure 2:**
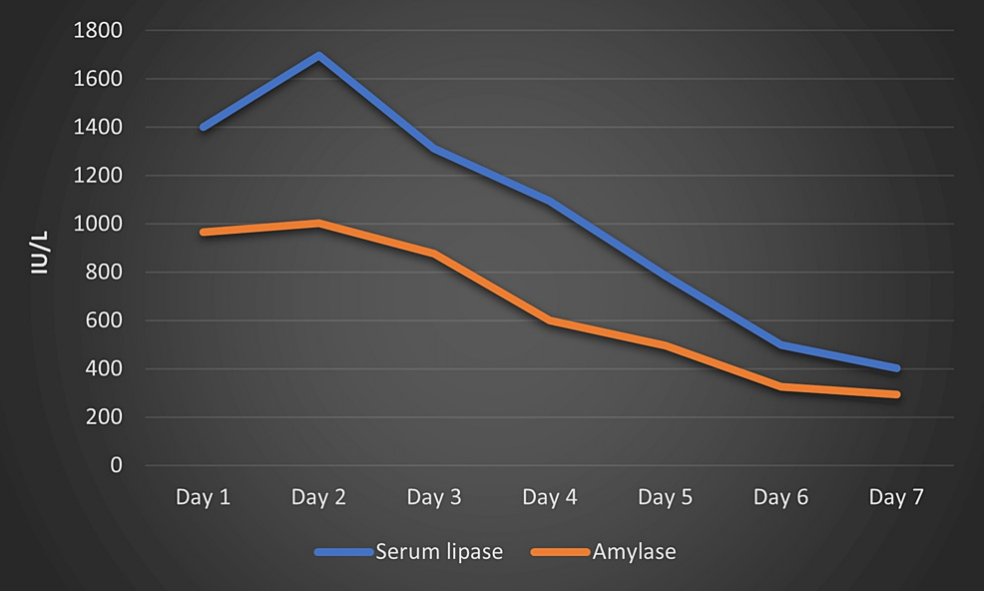
Graph demonstrating serum amylase and lipase level during hospital stay.

Immediately, levofloxacin was discontinued, and she was kept oral-free and was managed conservatively with bowel rest, intravenous fluids, appropriate analgesia, antiemetic, and proton pump inhibitor. For a urinary tract infection, she was started on fosfomycin. Over the next seventy-two hours, her symptoms started improving with the resolution of serum lipase and amylase level (Figure 2). She was discharged on day seven with a regular follow-up with her physician.

## Discussion

Drug-induced acute pancreatitis is estimated to account for 5% of all acute pancreatitis cases, and the global incidence is crossing 50-800 per one million adults [6]. Drug-induced acute pancreatitis is relatively rare and a diagnosis of exclusion after ruling out alcohol, gallstones, hypertriglyceridemia, and intervention [7]. Diuretics, valproic acid, and azathioprine are commonly associated with drug-induced acute pancreatitis. Many drugs have been listed as an etiology of acute pancreatitis, including antihypertensives, antifungal, antiviral, and antibiotics [8]. Levofloxacin-induced cute pancreatitis is uncommon, and the number of cases reported is few in numbers. We have tabulated the reported cases of drug-induced acute pancreatitis triggered by levofloxacin (Table 2).

**Table 2:** Reported cases of levofloxacin-induced acute pancreatitis. y: year, M: male, F: female, LVF: levofloxacin.

Author et al.	Age/Sex	Clinical presentation	Clinical condition for LVF use	Duration of LVF use (days)	Lab value (lipase, amylase)	Management	Outcome
Neto Goncalves et al. [9]	74y/M	Epigastric pain, nausea	Acute cystitis	5	Elevated	Drug withdrawal, conservative	Discharged
Jiménez et al. [4]	57y/F	Epigastric pain, malaise	bronchiectasis	2	Elevated	Drug withdrawal, conservative	Discharged
Rekhi et al. [10]	26y/F	Abdominal pain, vomiting	Acute pneumonitis	3	Elevated	Drug withdrawal, conservative	Discharged
Mennecier et al. [11]	31y/F	Abdominal pain, vomiting	Acute sinusitis	2	Elevated	Drug withdrawal, conservative	Discharged

Acute pancreatitis is manifested by elevated levels of serum amylase and lipase enzymes in the body. Laboratory findings should be concomitant with the histopathological findings of pancreatic tissue. Serum lipase levels more than two times normal are the most appropriate diagnostic marker for acute pancreatitis [5]. However, increased amylase levels are not diagnostic because the salivary glands also secrete the amylase enzyme, but the lipase enzyme is only secreted by pancreatic tissues [12]. The mechanism of drug-induced pancreatitis is the involvement of a toxic metabolite or a drug that either directly affects the biochemistry of the pancreatic cells or triggers an immune response against normal pancreatic tissue that further damages the structure and integrity of pancreatic tissues [13]. Both mechanisms result in necrosis of the pancreatic cells leading to acute pancreatitis. The lipid peroxidation pathway is an essential pathway behind both these mechanisms. It is also observed that reactive oxidative stress has a significant role in levofloxacin-associated nephrotoxicity. The pathophysiology includes an increased concentration of cellular adenosine triphosphate (ATP) and oxidative phosphorylation, leading to high production of free radicals inside the cells. This production can happen either directly or indirectly [14].

In our case, the patient developed acute pancreatitis shortly after initiating levofloxacin therapy, with no other identifiable causes present. The absence of gallstones, alcohol use, and recent biliary procedures supports the association between levofloxacin and pancreatitis. The clinical course of the patient improved following the discontinuation of levofloxacin and supportive care, indicating a drug-induced etiology. Physicians should rule out every possible cause to reach a definitive diagnosis [15]. 

## Conclusions

Levofloxacin-induced acute pancreatitis is a rare but potentially serious adverse drug reaction. The diagnosis is generally delayed as this is one of the uncommon causes of pancreatitis. Clinicians should be aware of this association when prescribing levofloxacin and closely monitor patients for any signs or symptoms of pancreatitis. Prompt recognition, withdrawal of the offending drug, and supportive care are essential for successfully managing and preventing complications associated with levofloxacin-induced acute pancreatitis. Further research is warranted to elucidate the exact pathophysiology and risk factors associated with fluoroquinolone-induced pancreatitis.
